# Hand Pose Recognition Using Parallel Multi Stream CNN

**DOI:** 10.3390/s21248469

**Published:** 2021-12-18

**Authors:** Iram Noreen, Muhammad Hamid, Uzma Akram, Saadia Malik, Muhammad Saleem

**Affiliations:** 1 Department of Computer Science, Lahore Campus, Bahria University, Islamabad 54000, Pakistan; 03-243182-026@student.bahria.edu.pk; 2 Department of Statistics and Computer Science, University of Veterinary and Animal Sciences (UVAS), Lahore 54000, Pakistan; muhammad.hamid@uvas.edu.pk; 3 Department of Information Systems, Faculty of Computing and Information Technology-Rabigh, King Abdulaziz University, Jeddah 21589, Saudi Arabia; Smalik1@kau.edu.sa; 4 Faculty of Engineering, King Abdulaziz University, Jeddah 21589, Saudi Arabia; msaleim1@kau.edu.sa

**Keywords:** hand posture, classification, deep learning, 2D CNN, multi stream, depth data

## Abstract

Recently, several computer applications provided operating mode through pointing fingers, waving hands, and with body movement instead of a mouse, keyboard, audio, or touch input such as sign language recognition, robot control, games, appliances control, and smart surveillance. With the increase of hand-pose-based applications, new challenges in this domain have also emerged. Support vector machines and neural networks have been extensively used in this domain using conventional RGB data, which are not very effective for adequate performance. Recently, depth data have become popular due to better understating of posture attributes. In this study, a multiple parallel stream 2D CNN (two-dimensional convolution neural network) model is proposed to recognize the hand postures. The proposed model comprises multiple steps and layers to detect hand poses from image maps obtained from depth data. The hyper parameters of the proposed model are tuned through experimental analysis. Three publicly available benchmark datasets: Kaggle, First Person, and Dexter, are used independently to train and test the proposed approach. The accuracy of the proposed method is 99.99%, 99.48%, and 98% using the Kaggle hand posture dataset, First Person hand posture dataset, and Dexter dataset, respectively. Further, the results obtained for F1 and AUC scores are also near-optimal. Comparative analysis with state-of-the-art shows that the proposed model outperforms the previous methods.

## 1. Introduction

Innovative technology has enabled us to communicate with computers and machines through touchless mechanisms in today’s fast-paced digital world. This mechanism involves postures such as waving hands, pointing fingers, and moving different body parts instead of touching screens, pressing switches, and raising voices [1]. This new paradigm of posture-based communication mechanics has evolved a new era of smart applications and challenges as well. This mechanism uses machine learning, pattern recognition, and computer vision technology. Posture recognition has concerns to the accurate identification of meaningful postures. Further, posture recognition has great significance in human–computer interfacing and intelligent systems. Posture recognition has various applications, e.g., smart surveillance, sign language recognition, human–robot interaction, vision based robotic surgery [2], handicapped medical assistance [3], TV control, gaming, and robot control [4]. The hand pose recognition problem has received the attention of researchers in the field of machine learning and computer vision [5] due to convenience and a wide range of applications. Hand-posture-based interfacing in applications has introduced convenience, flexible products, and efficiency for users to control devices without physical contact [6]. However, being an evolving domain, it confronts many issues and challenges. Traditional hand posture recognition methods began at the beginning of the 1980s [7]. Until recently, most posture recognition approaches were focused mainly on RGB intensity images. The main limitation in such RGB image datasets is that they are highly sensitive to illumination conditions, cluttered backgrounds, camera resolutions, and diverse points of view. This is problematic for segmentation, motion analysis, and detection of points of interest, and can perform well only in limited scenarios.

With the introduction of Kinect in 2010, easily available depth data has improved the domain. Firstly, with recent advances in depth camera technology, depth data can be acquired for much less cost. Secondly, depth data provide better information descriptions for the hand poses. Third, previous approaches are only effective with a limited number of gestures. Most of the work reported in the literature focuses on using conventional machine learning approaches with RGB data and depth data as well.

Recently, deep learning approaches have achieved state-of-art results as compared to conventional handcrafted feature-based techniques; however, they have also not investigated depth data much in comparison to RGB data. In recent years, CNNs have gained popularity and attention. Most of the CNN-based approaches involve 3D data representation of 2D depth images. Depth images are converted into 3D voxels to utilize 3D spatial information followed by an application of a 3D CNN for 3D hand pose classification. The methods using 3D point cloud-based inputs have performed well in capturing the geometric features of depth images; however, they suffer from complex data-conversion phases and also involve heavy parameter management, increasing the time complexity. Three-dimensional CNNs have complex network structure with a larger number of parameters and high computational cost. In the same way when a depth image is mapped to a 3D space, it may cause information loss or add unnecessary information (noise), resulting in errors. Therefore, 3D CNN-based methods not only waste convolution process calculations, but also move the neural network from learning effective features. Additionally, it is reported in the literature that multiple streams are helpful to improve recognition performance [8]. Further, little work is also reported with two-stream CNN using the optical flow method, but use of the optical flow method increases the process complexity [9]. Hand posture recognition remains a challenging problem and needs improvement. In this study, we designed a four-stream 2D CNN (2-dimensional convolutional neural network) to resolve the aforementioned issues. The main contribution of this study can be summarized as follows:

- The proposed a deep learning model comprises four parallel-stream 2D CNN for recognition of hand postures.
- The proposed model is lightweight compared to complex 3D CNN, which has a heavy set of parameters. Hyper parameters tuning using experimentation and multiple parallel streams enhance the performance of the proposed model.
- The proposed model outperforms existing state-of-the-art methods. An accuracy of 99.99%, 99.48%, and 98% is achieved using the Kaggle hand posture dataset, First Person hand posture dataset, and Dexter dataset, respectively.
- The evaluation of the proposed model on three different benchmark datasets independently makes it more robust and generalized.

The rest of the article is organized as follows. Section 2 describes related work. Section 3 explains the proposed approach. Section 4 presents the experimentation and results in discussion, which is followed by conclusion and potential future research directions in Section 5.

## 2. Related Work

The research community is active in the domain of hand pose recognition and a number of researchers have addressed this problem. Prominent work in the domain is described in this section. Zhu et al. [1] proposed a method to discuss the problem of HRI (human–robot interaction) and SAIL (smart assisted living) for disabled and elderly people. They used a neural network for posture spotting and HMM for context-based identification of posture. They collected data from the foot and waist joints. Cheng et al. [10] presented a survey on 3D posture estimation and presented a state-of-the-art analysis for 3D hand modeling, hand trajectory, continuous hand postures, and static hand posture estimation.

Plouffee et al. [11] proposed a method for static and dynamic posture recognition. They developed a natural posture UI (user interface) to track real-time hand postures using depth data of the ASL (American Sign Language) data set. They extracted the area of interest of the hand using a segmentation process with the assumption that the user is the closest object or entity in the scene to the camera. They improved scanning time on hand contour. They identified fingerprints using a k-curvature algorithm to recognize a person performing posture using the DTW (dynamic time warping) algorithm. They achieved a recognition rate of 92.5% for fifty-five static and dynamic postures.

Liu et al. [7] proposed a method for 3D posture recognition to accord the skeleton tracking of a person. They collected skeleton data from depth images generated through Kinect. Wu et al. [12] presented a method known as DDNN (deep dynamic neural network) for posture recognition using both depth and RGB data. They used a semi-supervised framework based on HMM for posture segmentation and DBN (deep belief network) to handle skeleton dynamics. Further, they adopted 3D CNN to fuse batches of depth and RGB data. Bao et al. [13] proposed a deep convolutional neural network method to classify postures without segmentation. Their method was able to classify seven types of hand postures in real-time and user-independent ways. Kumar et al. [14] proposed a robust position invariant SLR (sign language recognition) framework to observe occluded sign postures. Nunez et al. [5] used Kinect sensors and HMM to obtain and process skeleton data. They presented a method based on CNN and LSTM (long short-term memory) for posture and human activity recognition. They used 3D sequences of the full-body scan. Saha et al. [15] used an ensemble of tree classifiers with a bagging mechanism for an efficient two-person interaction detection system.

Supancic et al. [16] introduced a novel test set to perform the challenging segmentation of the active hand. Jun et al. [17] presented a vision-based hand posture recognition system to accord high security in an IoT (Internet of Things) application. The system was able to interact with users by recognizing postures in the captured images through a monocular camera installed on a terminal device. The first module of the system used an edge repair-based hand segmentation algorithm and the second module located the user’s position using an adaptive method. Avola et al. [18] used RNN (recurrent neural network) to train angles formed through the bones of fingers. Features were acquired through a LMC (leap motion controller) sensor and were selected based on hand joint movement. They achieved 96% accuracy on the ASL dataset and acquired more than 70% accuracy on the SHREC dataset. Tai et al. [19] proposed a many-to-many LSTM-based scheme for hand posture recognition. They applied maximum posteriori estimation on sensory data received by a gyroscope and also implemented a smartphone application to measure the performance. Mirehi et al. [20] presented a method based on fine-tuned inception V3 for static posture recognition from RGB-D data and obtained nearly 90% accuracy using ASL and NTU hand digit datasets. They also presented the SBU-1 dataset [21] that comprises multiple variations and deformation of hand postures.

Sanchez-Riera et al. [22] presented a method for posture recognition of multiple persons using the ICP (iterative closest point) algorithm. It is a minimization function that is initialized through parameters obtained through a dataset. Pinto et al. [23] used a combination of segmentation, polygonal approximation, contour detection, and morphological filters during preprocessing for better feature extraction followed by a CNN. Zhang et al. [24] proposed a robust feature descriptor based on path signature. They proposed the AOH (attention on hand) principle to identify single joint and pair joint features. They achieved an accuracy of 82% on the NVIDIA hand dataset. Hu et al. [25] presented a hand gesture recognition system using CNN with eight layers to control unmanned aerial vehicles (UAV). They achieved an average accuracy of 96%. However, the model is applicable only for non-scaled datasets. Okan et al. [26] also presented a CNN-based hand gesture recognition approach for video data. They applied a sliding window approach and evaluated the performance efficiency on two public datasets, NVIDIA, and EgoGesture. Their main contribution was to improve memory and computing requirements. Jinxian et al. [27] proposed an approach using PCA and generalized regression neural network (GRNN). They obtained classification results of hand gestures from the model and applied them further to extract human emotions with 95% accuracy. However, their approach was applicable to only nine static human gestures. Chen et al. [28] used CNN to recognize hand gestures through surface electromyography signals (sEMG). However, for classification, they used the traditional machine learning classification model. They trained the model on the Myo dataset and achieved an accuracy of 90%.

Kolivand et al. [29] acquired 96% accuracy on depth data of American Sign Language (ASL) by artificial neural network (ANN) and support vector machine (SVM) using radial basis function (RBF). Their main contribution was to devise a rotation invariant procedure of geometric feature extraction from hands received by depth camera. However, the limitation is that this scheme is manual. Similarly, Kapuściński et al. [30] trained near-neighbor and SVM methods using a static depth dataset of Polish Sign Language (PSL). They proposed a distance-based descriptor to recognize static gesture images. Though their method showed good performance but inherits all the limitations of manual procedure.

Human activities are categorized into group collaborations, interactions, actions, and gestures according to different complexity level. Warchoł et al. [31] proposed a bone pair descriptor and distance descriptor for skeletal data. Their method is beneficial as it is light and position invariant; however it is designed for ‘action’ recognition and not for hand ‘gesture’ recognition. Garcia et al. [32] presented a real-time segmentation method for hand gesture recognition using RGB frames. They demonstrated its performance on the IPN hand dataset comprising thirteen different gestures for touchless screen interaction. Sarma et al. [9] presented 2D CNN and 3D CNN using optical-flow-based motion template. The main issue with recent 2D or 3D approaches based on optical flow modalities is that they are computationally expensive and not suitable for real-time application due to increased complexity and computation cost. Though several researchers have addressed the hand posture recognition problem, very little work is reported using depth data in proportion to RGB data. Moreover, much of the work with depth data is focused either on action recognition or applies traditional machine learning methods, which are constrained to manual or handcrafted feature extraction. Manual procedures are undesirable because they are prone to errors, skill dependent, and time consuming, and involve tedious labor.

## 3. Proposed Model

Deep learning has gained immense popularity recently due to its ability of auto feature detection and high performance with image/video data. The true potential of deep learning is unleashed when the dataset is large. It is also reported in the literature that the use of multiple different streams can improve the performance in the image recognition accuracy [9]. We used 2D CNN with multiple parallel streams for posture classification. The proposed model was trained and tested using depth data and able to recognize with improved accuracy than state-of-the-art model methods. Description of datasets and the proposed model is provided in the following subsections.

### 3.1. Data Set Description

Three benchmark public research datasets were adopted to train and test the model. All three are static depth datasets. They are challenging and provide various types of hand gestures. Detail of each dataset is provided in this section.

A hand gesture recognition database was adopted from Kaggle [33], which is a public repository. It offers 10 different classes for hand gesture images for 10 digits (0–9). Each class is a collection of 2000 images representing different digits by hand gestures. Features are collected using LMC (leap motion controller).

The First Person [34] hand dataset consists of 105,469 frames in total with accurate hand pose and action categories, including annotations. This dataset consists of 45 hand action categories recorded in three different scenarios: kitchen, office, and social. This dataset was captured by an Intel RealSense SR300 RGB-D camera with 640 × 480 resolution for depth stream and 1920 × 1080 resolution for color stream [34].

The Dexter dataset [22] consists of seven classes using a multi-camera setup comprising five RGB cameras, one ToF (time-of-flight) camera, and one Kinect camera. RGB images were captured by Sony DFW-V500 cameras, whereas depth images were captured by Creative Interactive Posture cameras as ToF depth data sensors [22].

### 3.2. Data Preprocessing

**Figure 1:**
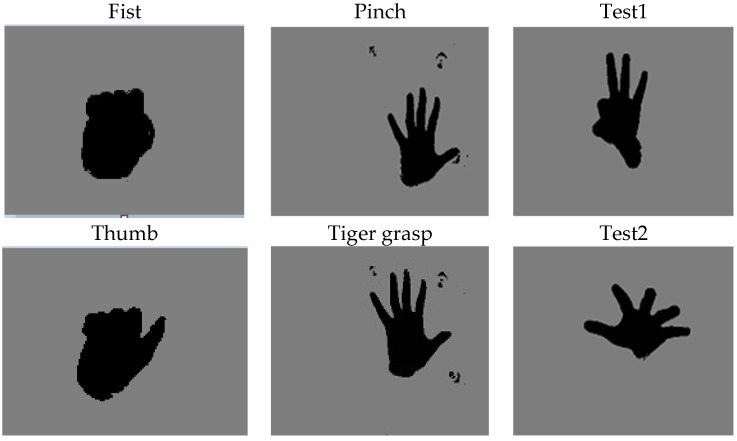
A few hand posture samples from selected hand posture datasets, (**left**) Kaggle Samples [33], (**middle**) Dexter Samples [22], (**right**) First Person Samples [34].

The model was trained using the low-cost depth datasets mentioned above, contrary to approaches using RGB images only. To provide better information descriptions for the hand poses, these datasets maintained RGB images by extracting hands from the depth data sample using view and color information associated with depth data. An augmented image data store function with preprocessing operations was used in MATLAB to transform batches of training and testing data. Randomized preprocessing operation was applied to prevent the network from memorizing the training images, hence avoiding overfitting. Then images were resized to 28 × 28 × 1, and color preprocessing function converted the images from RGB to gray. Finally, augmented images from the depth dataset were fed into the model for training. A few samples from each dataset are depicted in Figure 1. Table 1 shows the details of the hand posture datasets.

### 3.3. Internal Architecture of the Proposed Model

In 2D CNN, the convolutional kernel can process data in two directions, i.e., x and y to compute the convolutional output, which is actually generated in the shape of a 2D matrix. This makes it more suitable for image-based operations. We defined four parallel 2D CNN streams; each one received input samples from the dataset. Each stream followed the 2-D convolution process in parallel. Further, a batch normalization layer was added before pooling layers in order to enable independent learning by each layer in the network. Outputs of previous steps were concatenated and fed to the FC (fully connected) layer. In the end, SoftMax was applied for the final classification.

Our 2D CNN network consists of 21 layers with 23 connections. Layers are arranged in a parallel fashion. There are four convolutional layers and a total of three filters with filter size 5 × 5, keeping stride size ‘1’ with dilation factor ‘1’. The padding style is the same, weight learning rate is 0.001, bias learning rate is 1, and bias initializer is 0. In batch normalization layer configuration, epsilon is 0.00001, offset learn-rate factor is 1, scale learning rate factor is 1, offset is initialized with zeros, and scale initializer consists of ones. In the max-pooling 2D layer, we further used stride size {1} and pool size {5}.

In this model, we used an Adam optimizer to train the model. Moreover, we experimented using different initial learning rates, but our model performed best at 0.001. We tried different mini batch sizes for all three datasets, but we obtained the best results on 50, 100, and 50 mini batch sizes on the Kaggle, Dexter, and First Person datasets respectively. The network is optimized by minimizing the categorical cross-entropy (CCE) loss, defined as

(1) $$L_{CCE}=-\sum_{i}^{N}\sum_{c}^{C}\left(y_{i,c}\log\left(p_{i,c}\right)\right),$$

where *C* represents the number of classes, *N* shows the number of samples in the minibatch, *y_i,c_* is 1 if the class *c* is the ground truth class of the sample *I*, otherwise it would be 0. Similarly, *p_i,c_* shows the predicted class distribution over the *C* classes for the *i*th sample.

**Figure 2:**
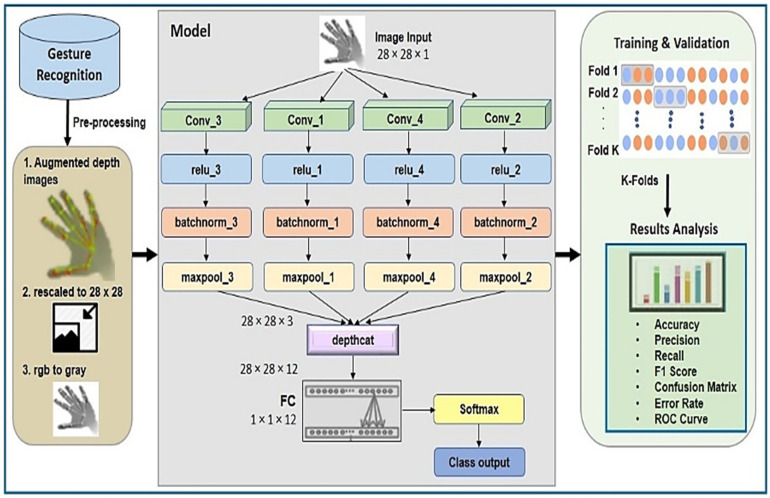
Proposed methodology.

The number of epochs is 25 for training purposes and each epoch consists of 552 iterations. We achieved the desired results with total of 13,800 iterations. We set the frequency as 30 iterations to validate our model. Figure 2 shows the proposed methodology. Table 2 describes the network parameters, and Table 3 shows the network architecture.

### 3.4. Experimental Setup

The programming language Python 3.7 was used to code the proposed approach. In our model’s experimental configuration, the experiment’s hardware environment was carried out on Intel MATL GPU + MATLAB 2019a. For the experimentation purpose, we used MATLAB GPU-based cloud offered by MathWorks. It enables researchers to train the models on a GeForce GTX 1080 machine without any expense for huge data. The computational power of MATLAB cloud servers enhances performance and saves computation time.

### 3.5. Training and Testing

We used the K-fold cross-validation strategy for training and validation to avoid overfitting and sampling bias. It also provides the opportunity for each observation to be used once for training as well as for validation. During model training, we performed 25 folds in leave-one-out order to avoid overfitting and to maintain accurate learning experience. Table 1 shows the training frames for each benchmark dataset, which are divided into 25 subsets to perform cross-validation. Average performance scores are used for final results.

### 3.6. Parameter Tuning for Performance

We compared the performance of the model by experimenting with different values of the optimizer, learning rate, mini batch sizes, number of epochs, number of iterations, and dropout. Table 4 shows the accuracy values of the model using different parameter values selected.

#### 3.6.1. Optimizer

Optimizers are used to tune the parameters of the model and to minimize the overall cost. Optimizers are usually categorized as gradient-descent optimizers and adaptive optimizers. This division is based on operational aspects. Optimizers manually tune the learning rate in the case of the gradient-descent algorithm whereas it automatically adapts in the case of the adaptive algorithm. Initially, we trained our model on both stochastic gradient descent with momentum (SGDM) and the Adam optimizer. We selected an Adam optimizer because our model learned features faster, converged rapidly, and also rectified vanishing learning rate with high variance.

#### 3.6.2. Learning Rate

Learning rate is a hyper parameter that actually defines the adjustment in the weights. It determines how fast or slow the training process moves towards optimal weights. Experimental observations showed that our model extracts features and classifies more accurately at the learning rate of 0.001. At lower values it improved feature learning.

#### 3.6.3. Mini Batch Size

Batch size is referred to as a total number of training samples per batch. It improves the effectiveness of training because the entire dataset cannot be passed to the neural network at once; therefore, a division of the dataset into batches or sets adds value. The amount of data included in each sub-epoch was adjusted through mini batch size. The mini batch size varied from 50 to 100 for different datasets and was adjusted to achieve the best throughput results for each dataset.

#### 3.6.4. Number of Epochs

Epoch is referred to as the complete pass of the entire dataset as forward and backward through the NN (neural network). We need to divide it into several smaller batches, however, because one epoch is too large to feed to the model at once. Using a single epoch can lead to under fitting, however, the higher the number of epochs, the higher the chances of training parameter readjustment, and the learning curve goes from under fitting to optimal and to overfitting. Our model was trained at an equal number of epochs, that is, 25, for all varying datasets to obtain a uniform distribution of trainable assets.

#### 3.6.5. Number of Iterations

Iterations are the number of batches or parts or sets needed to complete one epoch. The number of batches is equaled to the number of iterations for one epoch. In our model, it varied among datasets depending on the number of samples in a dataset.

#### 3.6.6. Dropout

Dropout removes certain neurons from the network. Each neuron in the network has a probability known as the dropout rate. Dropout improves the generalization capabilities of the network. Our model dropout rate was set to 0.5 so that features with a probability of less than 50% would be removed. Only strong features participated in classification.

## 4. Results and Discussion

**Figure 3:**
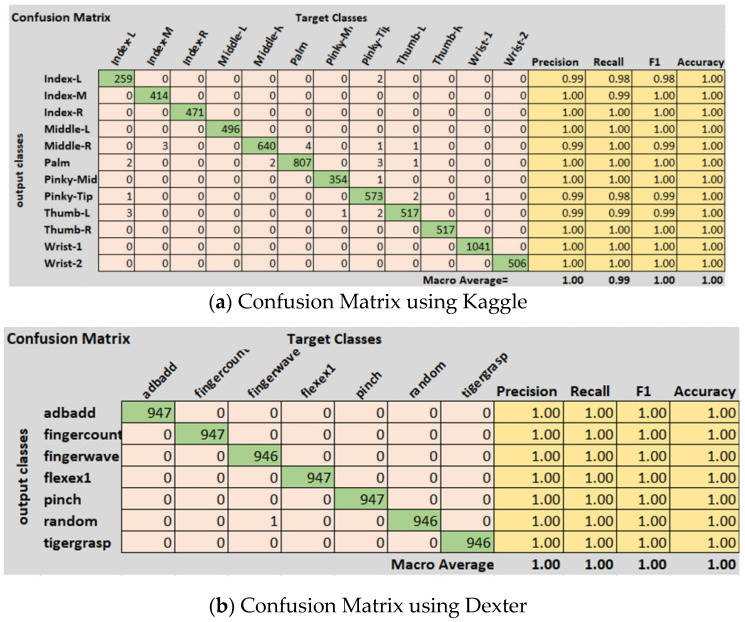

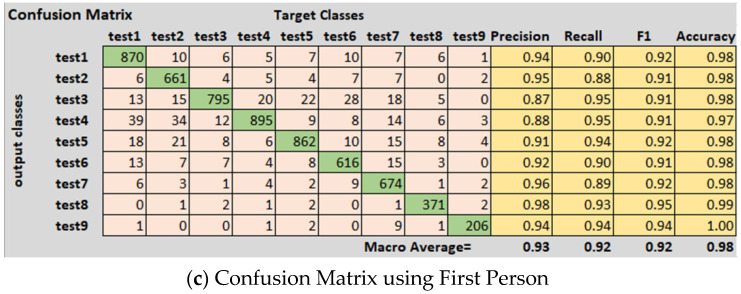
Confusion matrices of the performance results by the proposed model by using (**a**) Kaggle, (**b**) Dexter, and (**c**) First Person datasets.

The accuracy score is not the true representative of a model’s performance. Therefore, other metrics are also used to evaluate the performance. We calculated sensitivity, specificity, precision, and recall for all three datasets. Moreover, the ROC curve and area under the ROC curve, known as the AUC-score, were also calculated to measure the performance. The ROC curve is a tradeoff between sensitivity and precision and tells us about the true, false positive, and false positive rates. If it is closest to the diagonal, then the curve is not fine. A better output is indicated by classifiers that offer curves closer to the top-left corner. The closest the curve gets to the space of the ROC 45-degree diagonal, the less precise the model is. The performance metrics are defined in Equations (2)–(5). The summary of the performance results as per metrics defined in Equations (2)–(5) are shown in Table 5. The datasets are multiclass, therefore average values for all metrics are calculated using macro averaging, and are provided in Table 5. Figure 3 shows the confusion matrix and class wise detail of *Precision*, *Recall*, *Accuracy*, and *F*1 score of the proposed model for all three datasets.

(2) $$Recall=\frac{TP}{\left(TP+FN\right)}$$

(3) $$Precision\;=\frac{TP}{\left(TP+FP\right)}$$

(4) $$Accuracy=\frac{\left(TP+TN\right)}{\left(TP+TN+FP+FN\right)}$$

(5) $$F1=2*\frac{\left(Precision\;*\;Recall\right)}{\left(Precision+Recall\right)}$$

**Figure 4:**
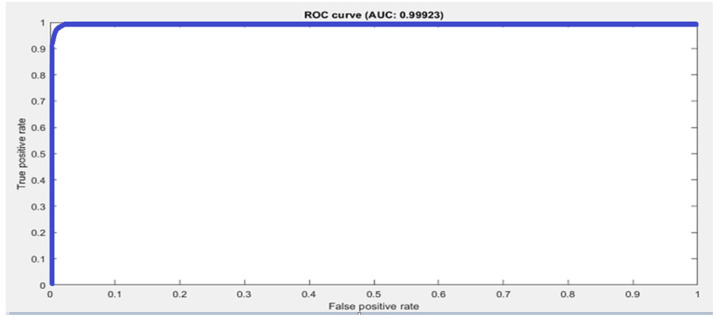
Macro average ROC curve and AUC score by the proposed approach using the Kaggle dataset.

The Kaggle hand posture dataset [33] contains 20 k frames in total; 13,375 frames for training and 6625 for testing are used. There are a total of 10 classes in the Kaggle hand posture dataset. Leap motion sensor was used to capture the dataset. In our model, we used an Adam optimizer with a starting learning rate of 0.001. Mini batch size in our model was set to 50 for the Kaggle dataset. A total of 25 epochs were used in our model, while 13,800 iterations were done in the proposed methodology. In our model, we used a total of 21 hidden layers and 23 connections. We used an image from the Kaggle dataset with the size 28 × 28 × 1 as an input to the model. We used a total of five 2D convolutional layers, five ReLU activation layers, five batch normalization layers, five max pooling layers, a fully connected layer, and a SoftMax activation function for posture classification. The proposed model achieved 99.99% accuracy using the Kaggle hand posture dataset. The calculated error rate for the Kaggle dataset was 0.01. The confusion matrix for the Kaggle dataset is shown in Figure 3a. The confusion matrix of 2D CNN architecture for Kaggle shows class wise true positive, false positive, true negative, and false negative information. Other useful metric results such as precision, recall, F1, and accuracy are also shown in Figure 3a. The ROC curve for the Kaggle dataset is shown in Figure 4. It shows true positive rate and false positive rate of the proposed model with a 1 and 0, respectively.

The Dexter hand dataset [22] contains 26k frames; 19,519 frames were used for training and 6481 frames were used for testing. The Dexter dataset consists of a total of seven classes. A Kinect sensor was used to capture the depth dataset, using five RGB cameras and one ToF camera. In our model, we used an Adam optimizer with a starting learning rate of 0.001. Mini batch size for the Dexter dataset was set to 100. A total of 25 epochs and 3090 iterations were used in our model. In our model, we used a total of 21 hidden layers and 23 connections. We used an image from the Dexter dataset with the size 28 × 28 × 1 as an input to the model. Using 2D CNN, we obtained a training accuracy of 99.91% and validation accuracy of 99.48% for the Dexter dataset. We calculated the error rate in the results obtained for the Dexter dataset, which was 0.52. 

**Figure 5:**
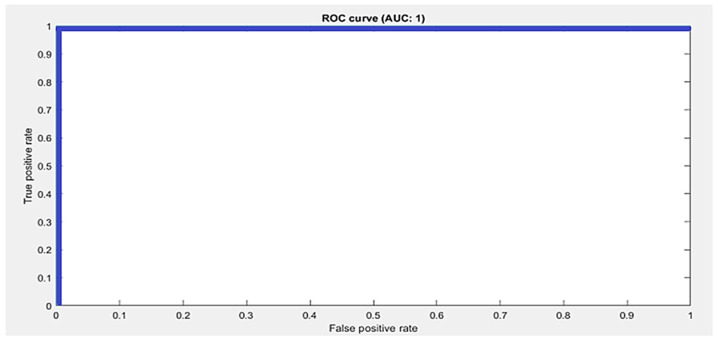
Macro average ROC curve and AUC score by the proposed approach using the Dexter dataset.

The confusion matrix of 2D CNN architecture for the Dexter dataset shows class wise true positive, false positive, true negative, and false negative information. Other useful metric results such as precision, recall, F1, and accuracy are also shown in Figure 3b. The ROC curve for the Dexter dataset is shown in Figure 5. It shows the true positive rate and false positive rate of the proposed methodology with a 1 and 0, respectively.

The First Person dataset [34] contains 105,469 frames in total, in which 98,842 frames are used for training and 6627 frames are used for testing. The First Person dataset consists of nine classes. Kinect sensor was used to capture the depth dataset. This dataset consists of 45 daily hand action categories. In our model, we used an Adam optimizer with a starting learning rate 0.001 and a mini batch size of 50 for First Person. A total of 25 epochs and 7550 iterations were used in our model. In our model, we used a total of 21 hidden layers and 23 connections. We used an image from the Dexter dataset with the size 28 × 28 × 1 as an input to the model. Using 2D CNN we obtained a validation accuracy of 98%. We calculated the error rate of the First Person dataset, which was 2.31.

**Figure 6:**
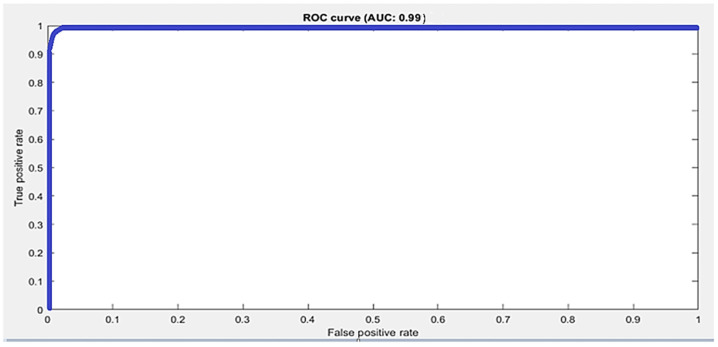
Macro average ROC curve and AUC score by the proposed approach using the First Person dataset.

**Figure 7:**
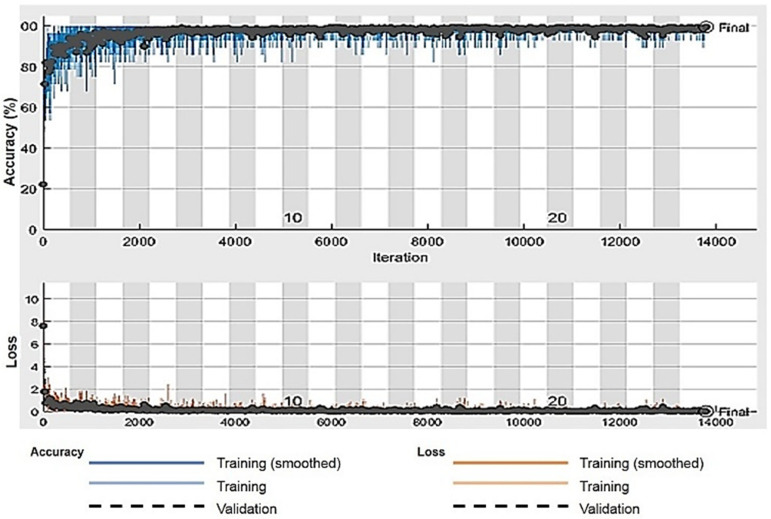
Plot of accuracy and loss for validation.

The confusion matrix of 2D CNN architecture for the Dexter dataset shows class wise true positive, false positive, true negative, and false negative information. Other useful metric results such as precision, recall, F1, and accuracy are also shown in Figure 3c. The ROC curve for the First Person dataset is shown in Figure 6. A training and validation accuracy-and-loss plot is shown in Figure 7 for the Kaggle dataset. It is evident from the plot that validation loss was minimal and there was not much gap between training and validation accuracies.

The results of the fine-tuned model are summarized and compared with state-of-the-art technique in Table 6. Sanchez-Riera et al. [22] attained 87% accuracy using CNN on the Dexter dataset. Garcia-Hernando et al. [34] attained 80% and 87% accuracy using Transition Forest and LSTM methods, respectively, for the First Person dataset. Tekin et al. [35] attained 88% accuracy using a hybrid approach of LSTM + MLP on the First Person dataset. Gadekallu et al. [36] attained 100% accuracy using CNN on the Kaggle hand posture dataset. Another recent two-stream 2D CNN-based approach by Sarma et al. [9] used an optical flow template scheme for static and dynamic hand gestures. In contrast, our proposed approach used four parallel streams for static datasets. Use of an optical flow scheme for a static dataset increases the computational cost and makes the process heavyweight. It is beneficial for a dynamic moving-hand scenario. Therefore, our approach avoids complexities of optical flow and is a lightweight method.

It is evident from the performance results that the proposed model outperformed the previous approaches. Moreover, it is also more generalized and robust than previous approaches. It was evaluated independently on three benchmark datasets. Hence, it is capable of recognizing a larger number of gestures than previous approaches.

## 5. Conclusions and Future Work

Hand posture recognition has received remarkable attention in recent years and plays a vital role in many fields such as robotics, gaming, surveillance, and HCI. Hand posture recognition using RGB intensity images has challenges due to lighting conditions and background clutter. However, depth image data have added value in the domain for better identification of postures than color images. In this study, 2D CNN with multiple parallel streams is used for posture identification using benchmark Kaggle, Dexter, and First Person datasets. Testing accuracy of the proposed method using the Kaggle dataset is 99.99%, using the Dexter dataset is 99.48%, and using the First Person dataset is 98%. Moreover, other evaluation matrices such as precision, recall, F1 score, AUC score, and root mean square error are also applied to measure performance. Comparison with other state-of-art approaches shows that our model has improved performance and is sufficiently robust. The future work plan is to extend the proposed approach as a multiple stream 3D CNN, and conduct ablation studies between 2D and 3D network behavior. Another future direction is to train the proposed model using hand gesture dataset of emergency/crisis vocabulary to develop an assistive application. Another future direction could be the hand posture estimation, which is the next level after gesture recognition. It requires a skeletal joint mapping process and identification of mean distance error for joint identification. Light, position, and rotation invariance capacity building is an open challenge to address by the community in this domain. Research in this direction will open new horizons of research and development due to the increased use of smart devices in the near future.

## Figures and Tables

**Table 1:** Details of hand posture datasets.

Title	Total Frames	Training Frames	Testing Frames	Number of Classes	Dimension
Kaggle [33]	20,000	13,375	6625	10	320 × 240
First Person [34]	105,469	98,842	6627	45	320 × 240
Dexter [22]	26,000	19,519	6481	7	320 × 240

**Table 2:** Details concerning the network parameters.

Network Parameters	Values
Total parameters	114,078
Trainable parameters	113,316
Non trainable parameters	762
Learning rate	0.001
Optimizer	Adam
Epochs	25
Iteration per epoch	552

**Table 3:** Network architecture details.

No	Name	Type	Activations	Learnable	Total Learnable
1	Image input 28 × 28 × 1 with ‘zerocente…	Image input	28 × 28 × 1	-	0
2	Conv_1 3 5 × 5 × 1 convolutions with stride…	Convolution	28 × 28 × 3	Weights 5 × 5 × 1 × 3 Bias 1 × 1 × 3	78
3	Conv_3 3 5 × 5 × 1 convolutions with stride…	Convolution	28 × 28 × 3	Weights 5 × 5 × 1 × 3 Bias 1 × 1 × 3	78
4	reLu_3 ReLU	ReLU	28 × 28 × 3	-	0
5	batchnorm_3 Batch normalization with 3 chan...	Batch normalization	28 × 28 × 3	Offset 1 × 1 × 3 Scale 1 × 1 × 3	6
6	maxpool_3 5 × 5 max pool with stride [1,1]	Max pooling	28 × 28 × 3	-	0
7	reLu_1 ReLU	ReLU	28 × 28 × 3	-	0
8	batchnorm_1 Batch normalization with 3 chan..	Batch normalization	28 × 28 × 3	Offset 1 × 1 × 3 Scale 1 × 1 × 3	6
9	maxpool_1 5 × 5 max pool with stride [1,1]	Max pooling	28 × 28 × 3	-	0
10	Conv_4 3 5 × 5 × 1 convolutions with stride…	Convolution	28 × 28 × 3	Weights 5 × 5 × 1 × 3 Bias 1 × 1 × 3	78
11	reLu_4, ReLU	ReLU	28 × 28 × 3	-	0
12	batchnorm_4 Batch normalization with 3 chan..	Batch normalization	28 × 28 × 3	Offset 1 × 1 × 3 Scale 1 × 1 × 3	6
13	maxpool_4 5 × 5 max pool with stride [1,1]	Max pooling	28 × 28 × 3	-	0
14	Conv_2 3 5 × 5 × 1 convolutions with stride…	Convolution	28 × 28 × 3	Weights 5 × 5 × 1 × 3 Bias 1 × 1 × 3	78
15	reLu_2, ReLU	ReLU	28 × 28 × 3	-	0
16	batchnorm_2 Batch normalization with 3 chan…	Batch normalization	28 × 28 × 3	Offset 1 × 1 × 3 Scale 1 × 1 × 3	6
17	maxpool_2 5 × 5 max pool with stride [1,1]	Max pooling	28 × 28 × 3	-	0
18	Depthcat Depth concatenation of 4 inputs	Depth concatenation	28 × 28 × 12	-	0
19	Fc 12 fully connected layer	Fully connected	1 × 1 × 12	Weights 12 × 9408 Bias 12 × 1	112,908
20	SoftMax	SoftMax	1 × 1 × 12	-	0
21	Classoutput crossentropyex with ‘index-L’…..	Classification output	-	-	0

**Table 4:** Experimentation results of parameter tuning for the Kaggle hand posture dataset.

Optimizer	Adam		
Learning Rate	0.01	0.001	0.0001
Batch Size	Accuracy%	Accuracy%	Accuracy%
25	91.57	99.60	98.78
30	92.27	99.99	99.32
60	98.51	99.58	97.74
90	98.43	99.62	98.99

**Table 5:** Summary of testing results of the proposed methodology with three datasets.

Dataset	*Accuracy*	*Precision*	*Recall*	*F*1	AUC	Mean Error
Kaggle [33]	99.99%	1	0.99	1	0.9992	0.01
Dexter [22]	99.48%	1	1	1	1	0.52
First Person [34]	98%	0.93	0.92	0.92	0.9900	2.31

**Table 6:** Comparison of the proposed model with state-of-art technique for all datasets.

Dataset	Previous Approaches				Proposed Approach
	Year	Reference	Technique	*Accuracy*	
Dexter [22]	2018	Sanchez-Riera et al. [22]	CNN	87%	99.48%
First Person [34]	2017	Garcia-Hernando et al. [34]	TF LSTM	80.69% 87.45%	98%
	2019	Tekin et al. [35]	LSTM + MLP	88.47%	
Kaggle [33]	2021	Gadekallu et al. [36]	CNN	100%	99.99%

## Data Availability

Data sharing is not applicable to this article.
